# Bioaccumulation and *in vivo* tracking of radiolabeled 4-nonylphenol in mice†

**DOI:** 10.1039/d3ra08743c

**Published:** 2024-03-14

**Authors:** Sajid Mushtaq, Soyeon Kim, Iqra Bibi, Ji Ae Park, Ji-ung Yang, Hyun Park, Jung Young Kim

**Affiliations:** a Division of Applied RI, Korea Institute of Radiological & Medical Sciences 75 Nowon-ro, Nowon-gu Seoul 01812 Republic of Korea sajidmushtaq@pieas.edu.pk +82-2-970-1977 +82-2-970-1660; b Department of Nuclear Engineering, Pakistan Institute of Engineering and Applied Sciences P. O. Nilore Islamabad 45650 Pakistan; c Korea National University of Science and Technology 217 Gajeong-ro, Yuseong-gu Daejeon 3411 Republic of Korea

## Abstract

4-Nonylphenol (4NP) is concerning due to its growing presence and endocrine-disrupting nature, raising concerns about its impact on health. In this study ^124^I-labeled 4NP was synthesized for *in vivo* tracing. Positron emission tomography imaging and biodistribution studies showed significant accumulation in various tissues after oral or intraperitoneal administration, emphasizing its intricate distribution and potential long-term effects, crucial for future risk assessments.

## Abstract 2

4-Nonylphenol, an emerging endocrine disruptor, raises health concerns. Radiolabeling and *in vivo* tracing of 4NP reveal significant tissue accumulation, emphasizing intricate distribution and potential long-term effects for risk assessments.

## Graphical abstract

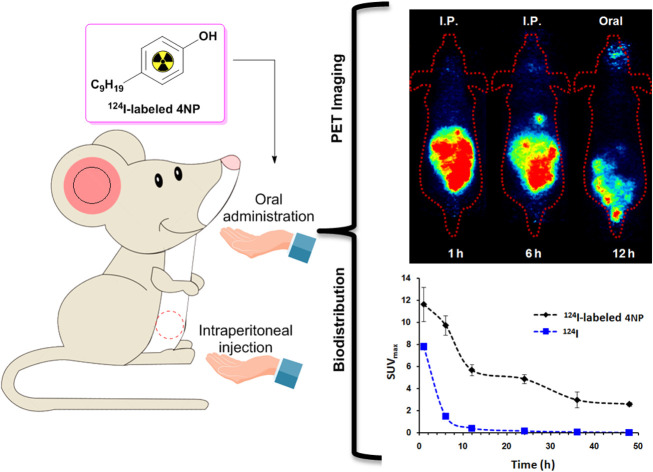

Mounting concerns have emerged recently regarding the potential health hazards posed by endocrine-disrupting chemicals (EDCs),^1^ which pervade our surroundings, infiltrate our food chain, and permeate numerous consumer products. Their insidious effects manifest through the disruption of hormone biosynthesis, metabolism, and functions, ultimately culminating in the disturbance of the body's harmonious homeostatic equilibrium and delicate reproductive processes.^2^ Nonylphenol (NP) is one of the most prominent Category 1 EDCs and is mainly derived from the degradation of nonylphenol polyethoxylates (NPEOs).^3^ NPEOs are extensively used in industries such as textile, paints, lubricants, pesticides, rubber, detergents, and food-packing materials.^4^ Moreover, NP has been detected in polyvinyl chloride (PVC), thereby leading to contamination of domestic water through PVC plumbing.^5^ After formation, NP exhibits remarkable persistence in the environment and can be detected in diverse environmental compartments, owing to its low solubility and resistance to biodegradation.^6^ NP has been detected worldwide in various environmental samples, including surface water, wastewater, ground waters, wastewater treatment plants, sediments, food, vegetables, dairy products, rivers, and lakes.^7^ Numerous studies have consistently supported the link between NP exposure and adverse effects on the endocrine, reproductive, immune, and central nervous systems,^8^ fatty liver disease, obesity, and cancer.^9^ Furthermore, NP exposure has been associated with reduced antioxidant enzyme levels in sperm, decreased testosterone levels in the blood, structural deformities in the testes, and Sertoli cell death due to abnormal apoptosis.^10^ Consequently, environmental exposure to NP and subsequent concerns for public health make it imperative to increase attention and scrutiny. To develop effective coping strategies, deeper understanding of the potential toxic effects and toxicokinetics behavior of NP is required. Currently, a limited number of *in vivo* and *in vitro* studies have investigated the adsorption, distribution, metabolism, and excretion (ADME) profile of NP in animals.^11^ Therefore, thorough characterization and comprehension of the toxicological dynamics of this EDC becomes essential.

Recently, advanced analytical techniques have been used to examine the ADME profile of NP or its analogs. These techniques include gas chromatography coupled with mass spectrometry (GC-ECNI/MS), Raman and Fourier-transform infrared spectroscopy (FTIR), electrospray ionization (ESI), atmospheric pressure chemical ionization (APCI) coupled with liquid chromatography-tandem mass spectrometry (APCI-LC-MS/MS), and ultra-performance liquid chromatography electrospray ionization-tandem mass spectrometry (UPLC-ESI-MS/MS).^12^ Although these methods have provided valuable insights into tissue distribution and *in vivo* behavior, several drawbacks remain. FTIR spectroscopy and MS exhibit inherent limitations such as low sensitivity, limited spatial and temporal resolution, and complex sample preparation requirements,^13^ while real-time imaging and *in vivo* EDC tracking are not available. An alternative and more efficient method for investigating the *in vivo* fate of EDCs is radiolabeling with a suitable radioisotope and subsequent tracking. Furthermore, it allows for noninvasive imaging of radiolabeled toxic compounds in specific areas of interest using single positron emission computed tomography (SPECT) or positron emission tomography (PET).^14^

In this study, we propose an innovative strategy whereby, 4-nonylphenol (4NP) was radiolabeled using iodine-124 (^124^I, *T*_1/2_ = 4.18 d) for the first time, enabling us to examine its *in vivo* behavior. The primary objective of this study was to gain insights into the intricate dynamics of 4NP in the living organism through PET imaging and biodistribution study.

## Result and discussion

**Scheme 1:**
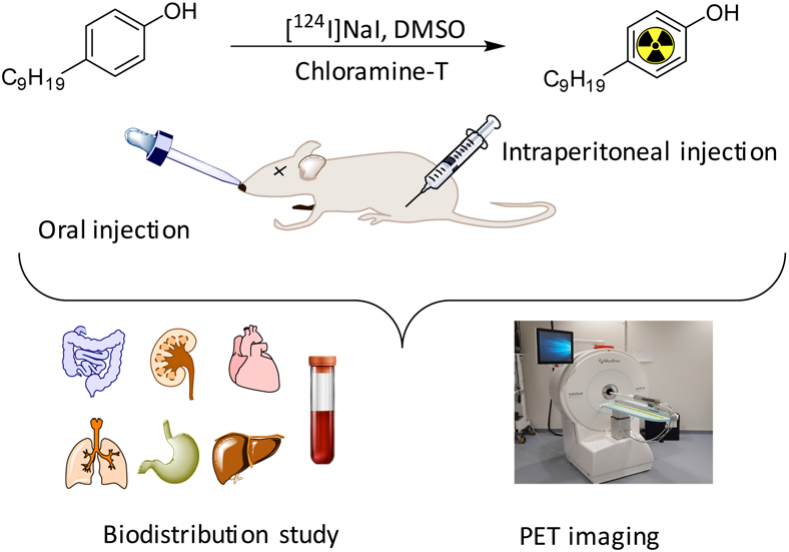
Schematic illustration showing radioiodination of 4-nonylphenol (4NP) and *in vivo* studies.

**Fig. 1:**
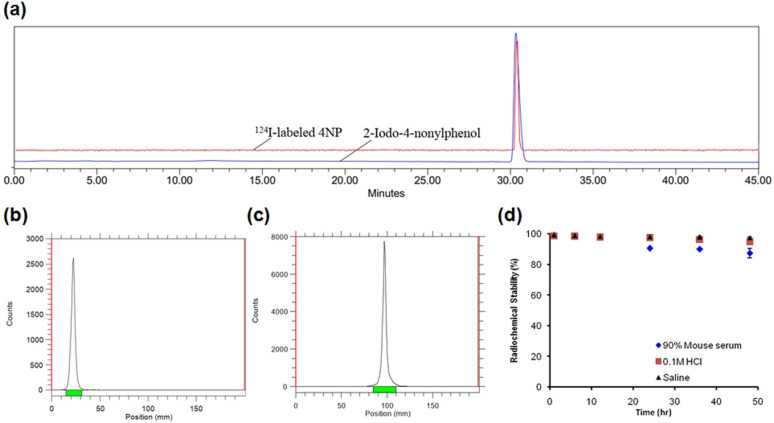
Characterization of ^124^I-labeled 4NP. (a) Radio- and UV-vis HPLC spectra of purified ^124^I-labeled 4NP and 2-iodo-4-nonylphenol, respectively, (b) radio-TLC spectrum of free radioiodine in ethyl acetate (c) radio-TLC spectrum of purified ^124^I-labeled 4NP in ethyl acetate, (d) *in vitro* stability of purified ^124^I-labeled 4NP in various media.

The synthesis of iodinated 4NP (2-iodo-4-nonylphenol) followed Scheme 1 and was comprehensively characterized using NMR (Fig. S1 and 2†) and mass spectrometry. This compound served as the basis for the identification and characterization of its radioiodinated counterpart, ^124^I-labeled 4NP. The synthesis of 2-iodo-4-nonylphenol encompassed a process employing NaI in the presence of chloramine-T as an oxidizing agent, with the reaction subsequently quenched using sodium metabisulfite. Purification was accomplished through preparative HPLC, with retention time observed at 30 min. A similar procedure was employed for the synthesis of ^124^I-labeled 4NP. The radiochemical yield for the synthesis of ^124^I-labeled 4NP was 130 MBq (95%), achieved within a 30 min interval. The crude product underwent purification *via* preparative HPLC, resulting in high radiochemical purity (>99%, *n* = 4) (Fig. 1a–c).

**Fig. 2:**
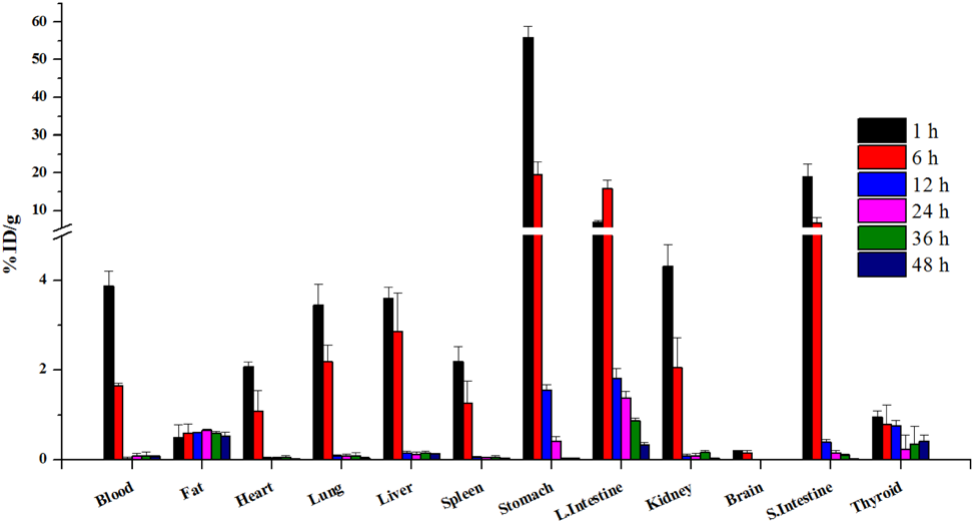
Biodistribution data of ^124^I-labeled 4NP after oral administration (*n* = 4).

To confirm stability, the radioiodinated compound was incubated in mouse serum (90%) and 0.1 M HCl (pH = 2) or saline for 48 h. ^124^I-labeled 4NP showed high stability (Fig. 1d) and more than 90% radioactivity remained intact up to 36 h, as determined using HPLC or radio-TLC. Nonylphenol is a heterogeneous mixture of compounds in which alkyl side chains, whether linear or branched, are attached to the phenolic ring. The study was conducted under the assumption that the pharmacokinetics of branched- and linear-chain phenols are closely analogous, as these phenolic compounds share physiochemical properties. This assumption was made because of the impracticality of performing pharmacokinetic experiments on a random mixture of branched-chain NPs.^15^ In the initial experiment, ^124^I-labeled 4NP was formulated in 10% ethanol solution in saline and administered orally to six groups of mice. Biodistribution data were collected at various time points post administration (1, 6, 12, 24, 36, and 48 h) and expressed as the percentage of injected dose per gram of organ or blood (%ID per g). As depicted in Fig. 2, a substantial concentration of the radioiodinated tracer was observed, which exceeded 50% in the stomach.

Interestingly, adsorption of ^124^I-labeled 4NP was rapid, followed by distribution to other organs, including small intestine (19.02 ± 0.02 %ID per g), large intestine (7.02 ± 0.27 %ID per g), kidneys (4.30 ± 0.48 %ID per g), liver (3.59 ± 0.24 %ID per g), and lungs (3.43 ± 0.47 %ID per g) 1 h post administration.

Notably, radioactivity signals were also detected in the bloodstream (3.86 ± 0.34 %ID per g) 1 h postinjection; these subsequently decreased to 1.63 ± 0.06 and 0.09 ± 0.03 %ID per g after 6 and 12 h, respectively. The concentration of the compound was also reduced in the liver, reaching 0.11 ± 0.02 %ID per g 48 h postinjection. These findings suggest that ^124^I-labeled 4NP was initially absorbed in the small intestine, gradually entered the bloodstream, and was subsequently distributed to various organs. Additionally, the radioiodinated compound was metabolized and excreted *via* the kidneys. An observable reduction in radioactivity signals in the stomach was recorded, decreasing to 19.58 ± 3.33 %ID per g after 6 h. During the same time frame, the highest concentration of the compound was detected in the large intestine (15.86 ± 2.19 %ID per g).

After 12 h, a significant portion of the compound localized predominantly in the large intestine (1.80 ± 0.21 %ID per g), with a substantial amount of ^124^I-labeled 4NP still present in the stomach (1.54 ± 0.12 %ID per g). Finally, at the 48 h postinjection mark, ^124^I-labeled 4NP was primarily detected in the large intestine (0.33 ± 0.05 %ID per g), liver (0.11 ± 0.02 %ID per g), and adipose tissue (fat) (0.62 ± 0.09 %ID per g) (Table S1†).

**Fig. 3:**
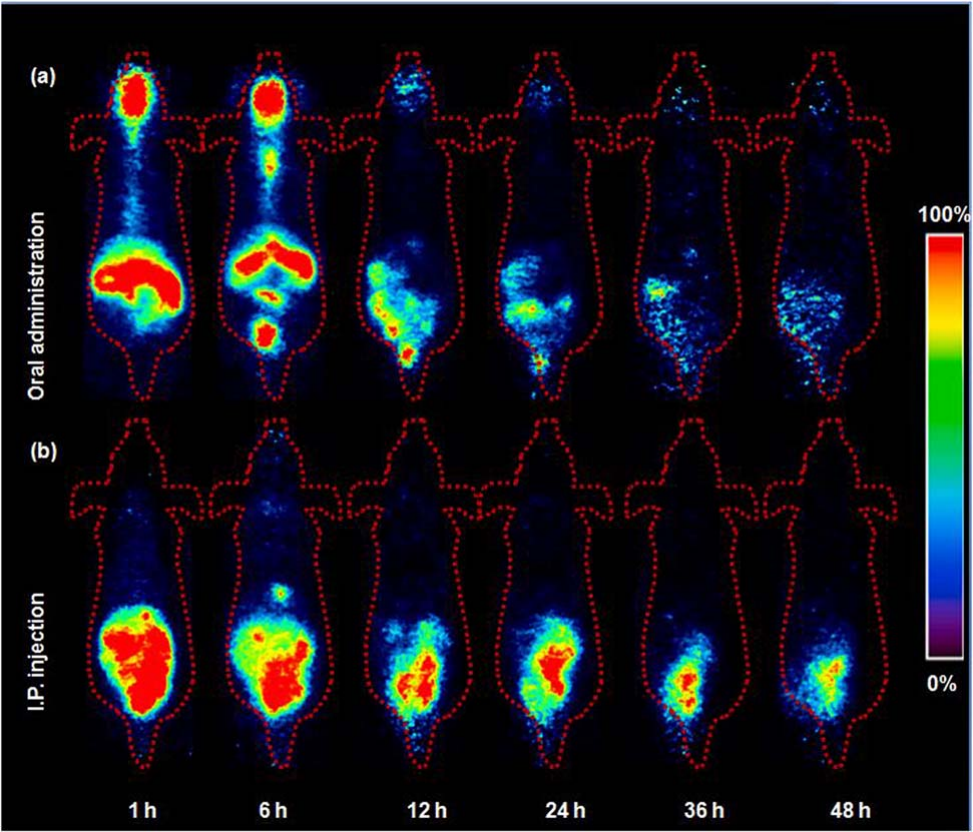
Representative PET images of ^124^I-labeled 4NP acquired using 6 weeks-old ICR mice. (a) Oral and (b) intraperitoneal (I.P.) injection (*n* = 3).

PET imaging data corroborated the observed absorption profiles (Fig. 3a). One hour postinjection, a substantial concentration of ^124^I-labeled 4NP was detected in the stomach, large intestine, and small intestine. Additionally, the radioiodinated compound persisted in the gastrointestinal tract (GI tract) throughout the designated time intervals, consistent with the biodistribution data. In a control experiment, free radioiodine (^124^I) was orally administered to mice (*n* = 3) and PET images were acquired under analogous conditions (Fig. S3a†). Radioiodine initially remained in the stomach, but, notably, elevated radioactivity signals were discernible in the thyroid gland 12, 24, 36, and 48 h postinjection as depicted in SUV_max_ data (Fig. S4†). The biodistribution outcomes following oral administration were in close agreement with those reported previously. Specifically, our findings corroborate the observations of Liu *et al.*,^16^ who documented a comparable biodistribution profile with 4NP predominantly absorbed in the large intestine, liver, and kidneys following oral administration. This consistency in distribution patterns is further supported by the rapid absorption of 4NP in the GI tract observed by Doerge *et al.*^17^ in their studies involving oral administration in rats. It is noteworthy that our present study employs a distinct and advanced methodology, utilizing radiolabeling technology coupled with positron emission tomography (PET) imaging. This innovative approach not only reinforces the concordance with previously reported biodistribution profiles but also extends our understanding by providing a dynamic and real-time assessment of 4NP distribution in various tissues over a comprehensive time range.

Previous research has indicated that sustained exposure to 4NP in female rats during pregnancy leads to elevated levels of pro inflammatory cytokines in the intestine, underscoring the potential adverse effects of 4NP on GI health.^18^ In another study, continuous oral exposure to 4NP in mice resulted in inflammation in both the body and stomach.^19^ Oral administration of ^124^I-labeled 4NP clearly demonstrated the relatively slow adsorption and extended accumulation of 4NP, which has the potential to induce damage to the GI organs.

**Fig. 4:**
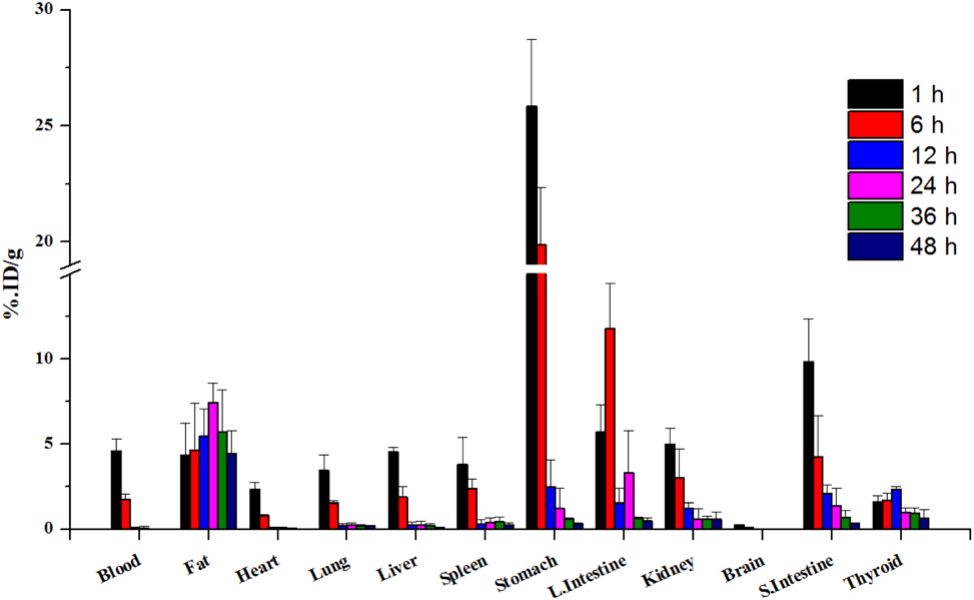
Biodistribution data of ^124^I-labeled 4NP after intraperitoneal injection (*n* = 4).

To obtain more detailed insights into the absorption of 4NP in the body, ^124^I-labeled 4NP was administered to the six groups of mice *via* I.P. injection and biodistribution data were collected at the various time points. ^124^I-labeled 4NP rapidly absorbed into the bloodstream and quick distribution throughout the body. One hour postinjection, we observed notable accumulation in the stomach (25.84 ± 2.81 %ID per g), small intestine (9.87 ± 2.52 %ID per g), large intestine (5.72 ± 1.58 %ID per g), liver (4.50 ± 0.27 %ID per g), and adipose tissue (4.35 ± 1.87 %ID per g) (Fig. 4). After 6 h, the radioactivity signals diminished in the stomach (19.77 ± 2.46 %ID per g), while a continuous increase was observed in the large intestine (11.78 ± 2.62 %ID per g) and adipose tissues (4.62 ± 2.75 %ID per g) (Table S2†). These results suggest absorption by the adipose tissue and elimination through the GI tract. Accumulation was highest in the adipose tissue (7.42 ± 1.16 %ID per g) 24 h postinjection, with high concentrations maintained 36 and 48 h postinjection (5.73 ± 2.45 and 4.42 ± 1.38 %ID per g, respectively). At the final time point, the radioactivity signals in the stomach and large intestine were low, whereas accumulation of ^124^I-labeled 4NP was high in the adipose tissue.

The higher accumulation of ^124^I-labeled 4NP in the adipose tissue can be attributed to the greater affinity of the compound for lipids.^20^ Numerous reports have demonstrated elevated concentration of 4-octylphenol (OP) and 4NP in the adipose tissue of both men and women following continuous exposure to these chemicals.^21^ In addition, Müller *et al.*^22^ reported rapid accumulation of NP in the GI tract and lipid compartment of the human body a few hours after oral administration. The increased accumulation in adipose tissue after I.P. injection can be attributed to several factors, including the potential for direct diffusion of the radiolabeled compound into this more lipophilic organ. The I.P. route may offer a more direct and rapid access to adipose tissue due to the proximity of the peritoneal cavity, allowing for enhanced absorption and distribution into adipose compartments. We also recognize that differences in absorption kinetics and first-pass metabolism between oral and I.P. routes may contribute to the observed variations in tissue distribution. PET imaging after I.P. administration was conducted under conditions similar to those described above, yielding results consistent with the biodistribution data. Initially, the radioiodinated compound was observed in the stomach, small intestine, and large intestine (Fig. 3b). Radioactivity signals in the lower abdominal area were detected 24, 36, and 48 h post-I.P. injection, indicating accumulation of radioactivity in the adipose tissue during these time intervals. In the control experiment with the free radioiodine, the PET images revealed rapid clearance of radioactivity from the abdominal area (Fig. S3b†). High radioactivity signals were detected in the thyroid gland 12 h post-I.P. injection, which persisted until the final time point similar trend was observed in Fig. S5.†^124^I-labeled 4NP remained stable throughout the experimental study and provided valuable information *in vivo*.

The biodistribution study of ^124^I-labeled 4NP revealed distinct patterns of accumulation in various tissues following oral and I.P. administration. After oral administration, 4NP distributed itself prominently among the GI tract, liver, and kidneys. Conversely, I.P. injection resulted in accumulation in adipose tissue, stomach, small intestine, large intestine, and other organs. Given the known endocrine-disrupting nature of 4NP, its potential health implications are significant. Although the complete characterization of 4NP's toxicity is ongoing, it is associated with adverse reproductive effects, including changes in the weights of testes and epididymis, alterations in sperm motility, viability, count, and concentration.^23^ Furthermore, 4NP induces oxidative stress, increases the incidence of various cancers (*e.g.*, urinary bladder, lung, kidney, liver, and thyroid gland), and contributes to DNA damage and apoptosis.^24^ The structural similarity of 4NP to endogenous estrogens suggests its potential to interfere with reproductive system function. Extensive studies have highlighted its adverse effects on the immune and digestive systems, with both animals and humans susceptible to damage due to 4NP's lipophilicity and persistence in the environment.^25^ Chronic exposure to low concentrations of 4NP raises concerns about its long-term toxicity, with confirmed impacts on neurotoxicity, liver toxicity, immunotoxicity, and developmental effects on brain tissue. These diverse toxic effects underscore the complex and multifaceted health implications associated with 4NP exposure. Further research is essential to comprehensively understand the potential risks and establish regulatory measures to mitigate the adverse effects of 4NP on both human and environmental health.

The methodology used in the present study offers several advantages, such as easy and straightforward sample preparation and quantitative assessment of ^124^I-labeled 4NP distribution in the blood and organs. PET imaging using ^124^I-labeled 4NP offers a comprehensive perspective of 4NP dispersion across the body, capturing dynamic processes and unveiling metabolic functions without invasive procedures. This systematic approach enriches our understanding of the characteristics of 4NP, its systemic spread, and metabolic behaviors. ^124^I-labeled 4NP differs structurally and chemically from 4NP but substitution of one hydrogen atom with an iodine atom does not likely alter significantly the pharmacokinetics in mouse tissues. This expectation is grounded on previous studies where numerous small molecules (drugs) were successfully labeled with radioiodine, demonstrating comparable biodistribution patterns and uptake in various organs and tumors.^26^

## Conclusion

In conclusion, radioiodinated analogue of 4NP (^124^I-labeled 4NP) was synthesized and administered to an animal model through diverse routes to facilitate physiologically based pharmacokinetic analyses. ^124^I-labeled 4NP was synthesized with a high radiochemical yield and purity. ^124^I-labeled 4NP accumulated in the stomach, large intestine, small intestine, and adipose tissue after oral administration and in the GI tract and adipose tissue for several hours' post-I.P. injection. PET imaging and biodistribution data encompassed critical parameters such as absorption, blood pharmacokinetics, excretion patterns, and tissue distribution. The application of such methodology provides valuable insights into potential adverse effects on the human population.

## Ethical statement

All animal experiments were approved by the KIRAMS Committee for Animal Welfare and were conducted in full compliance with the guidelines outlined in the Korean Animal Protection Law.

## Conflicts of interest

There are no conflicts to declare.

## Supplementary Material

RA-014-D3RA08743C-s001
