# Supplementary material for: Bioaccumulation and in vivo tracking of radiolabeled 4-nonylphenol in mice
Source: RSC Adv. 2024 Mar 14;14(12):8578–82. doi: 10.1039/d3ra08743c (PMC10938378; doi:10.1039/d3ra08743c)
Supplement: RA-014-D3RA08743C-s001 [file RA-014-D3RA08743C-s001.pdf]

Electronic Supplementary Material (ESI) for RSC Advances.
This journal is © The Royal Society of Chemistry 2024

Bioaccumulation and *in vivo* tracking of radiolabeled 4-Nonylphenol in mice

Sajid Mushtaq[a,b,*,‡], Soyeon Kim[a,‡], Iqra Bibi[a,c], Ji Ae Park[a,c], Ji-ung Yang[a], Hyun Park[a], Jung Young Kim[a]

[a]*Division of Applied RI, Korea Institute of Radiological & Medical Sciences, 75 Nowon-ro, Nowon-gu, Seoul 01812, Republic of Korea.*
[b]*Department of Nuclear Engineering, Pakistan Institute of Engineering and Applied Sciences, P. O. Nilore, Islamabad 45650, Pakistan*
[c]*Korea National University of Science and Technology, 217 Gajeong-ro, Yuseong-gu, Daejeon 3411, Republic of Korea.*

‡ Sajid Mushtaq and Soyeon Kim contributed equally to this work

*__Corresponding Authors:__ Professor Sajid Mushtaq

Division of Applied RI, Korea Institute of Radiological & Medical Sciences (KIRAMS), 75 Nowon-ro, Nowon-gu, Seoul 01812, Republic of Korea
Telephone number: +82-2-970-1660
Fax number: +82-2-970-1977
E-mail address: sajidmushtaq@pieas.edu.pk

Material and Methods

All reagents and solvents, including 4-nonylphenol (4NP), analytical standard, chloramine-T hydrate, sodium metabisulfite, sodium iodide, dimethylformamide (DMF), acetonitrile (ACN), trifluoroacetic acid (TFA), and dimethyl sulfoxide (DMSO) were purchased from Merck Korea (Seoul, Republic of Korea).Sep-Pak C18 cartridges were purchased from Waters, United States. The ^{1}H and ^{13}C nuclear magnetic resonance (NMR) spectra were obtained using Bruker Avance III HD NMR spectrometer at 600MHz. DMSO-d_6 was used as the solvent. The radioactive and nonradioactive 4NP analogs were analyzed and purified using the Waters Alliance semi-preparative high-performance liquid chromatography (HPLC) system equipped with gamma detector and XTerra Prep RP_{18} columns (10 × 250 mm, 10.0µm).The solvent compositions A (0.1% trifluoroacetic acid in water) and B (0.1% trifluoroacetic acid in acetonitrile) were used for the HPLC experiments. The method involved a linear gradient approach with a flow rate of 4 mL/min. The gradient was programmed as follows: 0–3 min (95% A/5% B), 3–20 min (20% A/80% B), 20–30 min (5% A/95% B), 30–40 min (95% A/5% B), and 40–45 min (5% A/95% B).The *in vitro* stability of the radiolabeled compound was monitored using an Eckert & Ziegler radio-thin-layer-chromatography (TLC) scanner (model: AR-2000) and HPLC system. The biodistribution study was performed on 6-week-old ICR mice, supplied by DooYeol Biotech (Seoul, Republic of Korea). The radioactivity accumulated in each organ was quantified using the PerkinElmer automatic gamma counter (model: 2480 automatic gamma counter).PET images were acquired using the Mediso nanoScan PET system. All animal experiments were approved by the KIRAMS Committee for Animal Welfare and were conducted in full compliance with the guidelines outlined in the Korean Animal Protection Law.

***Synthesis of iodinated* 4NP (2-iodo-4-nonylphenol)**

Sodium iodide (17mg, 0.113 mmol) and chloramine-T hydrate (25.87 mg, 0.113 mmol) were added sequentially to a stirred solution of 4NP (20 mg, 0.090 mmol) in DMSO (5 mL). The reaction mixture was stirred for 3h at room temperature, the progress of the reaction was monitored using HPLC, and sodium metabisulfite (40mg, 0.226 mmol) was added to quench the reaction. The compound was purified using HPLC and characterized using NMR (Figure S1 and S2) and HRMS. 2-Iodo-4-nonylphenol was obtained in moderate yield (18mg, 57.48%),^{1}H NMR (600 MHz, DMSO-d_6) δ 0.79–0.82 (t, 3H), 1.18–1.23 (m, 12H), 1.42–1.45 (q, 2H),2.36–2.39 (t, 2H) 6.72–6.74 (d, 2H), 6.94–6.95 (d, 2H), 7.41 (s, 1H), 9.98 (s, 1H).^{13}C NMR (600 MHz, DMSO-d_6) δ 14.22, 22.36, 28.76, 28.94, 29.08, 29.21, 31.39, 31.53, 33.86, 84.55, 115.02, 129.57, 135.22, 138.39, 154.75.HRMS ($[M+H]^+$) calculated for $C_{15}H_{24}IO^+$: 347.0827; found 347.0795.

Production of ^{124}I

Radioiodine (^{124}I, 370MBq/100μL), supplied by KIRAMS, Seoul Republic of Korea, was provided in a 0.1N solution of sodium hydroxide.

Synthesis of radioiodinated 4NP (^{124}I-labeled 4NP)

A solution of 4NP (2mg, 0.009 mmol) in DMSO (200μL) was prepared. Sequentially, acetic acid (50μL) and chloramine-T (1.5mg, 15μL) were added to this solution. To initiate the reaction sequence for radioiodination, a solution of [^{124}I]NaI (185 MBq) in 0.1 M NaOH (150μL) was introduced into the reaction mixture, which was then incubated at room temperature for 30 min.

To terminate the reaction, a solution of sodium metabisulfite solution (20mg, 20μL) was added. The crude reaction product was subjected to purification using HPLC. The purified product was diluted with 10 mL of deionized water and loaded into a C18 Sep-Pak cartridge, which was previously conditioned with ethanol (6 mL) followed by deionized water (12 mL). Subsequent elution of the trapped product was accomplished using absolute ethanol (500 μL), and the eluted solution was then diluted with saline in preparation for further *in vitro* and in *vivo* experiments.

In vitro **stability of** ^{124}**I-labeled 4NP**

For assessment of *in vitro* stability, a sample of purified ^{124}I-labeled 4NP (740 kBq, 10μL) was incubated in 500μL of mouse serum (90%) and 0.1M HCl or saline, at room temperature for 48 h. Stability analyses were conducted at specific intervals (1, 6, 12, 24, 36, and 48h) using either a radio-TLC scanner or a HPLC system. Each experiment was replicated thrice to ensure reliability and consistency of results

PET imaging of ^{124}**I-labeled 4NP**

Comprehensive *in vivo* studies were carried out on a state-of-the-art PET system. The ^{124}I-labeled 4NP, at a concentration of 5.55 MBq/100 μL in saline medium, was introduced into mice via the oral or I.P. route. Subsequent PET imaging was conducted under 2% isoflurane at multiple temporal intervals.

Biodistribution study of ^{124}**I-labeled 4NP**

For the biodistribution analyses, a ^{124}I-labeled 4NP concentration of 740 kBq/100 μL in saline matrix was maintained. The radiolabeled compound was introduced systemically via oral or I.P. administration. At specified intervals, four mice per time point were euthanized, and their

respective organs and blood samples were harvested. The radiotracer accumulation within each organ was quantified using an automated gamma counter, and the outcomes were expressed as a percentage of the injected dose per gram (%ID/g) of the organ.

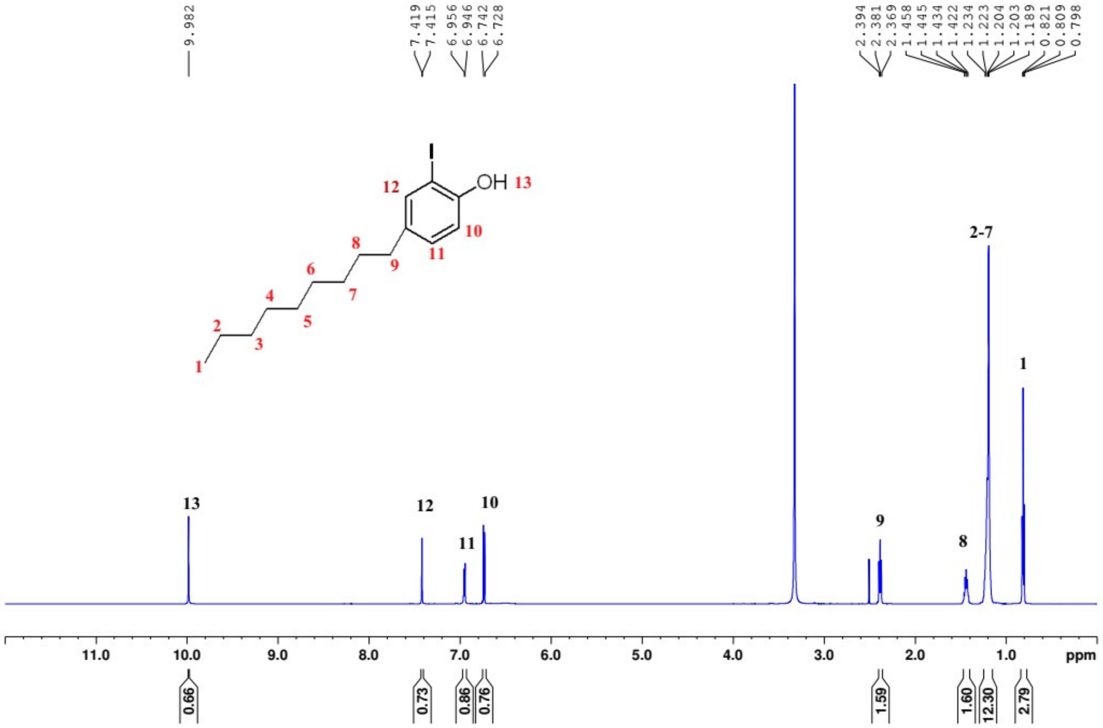

Figure S1: ^{1}H NMR spectrum of 2-iodo-4-nonylphenol in DMSO-*d6*

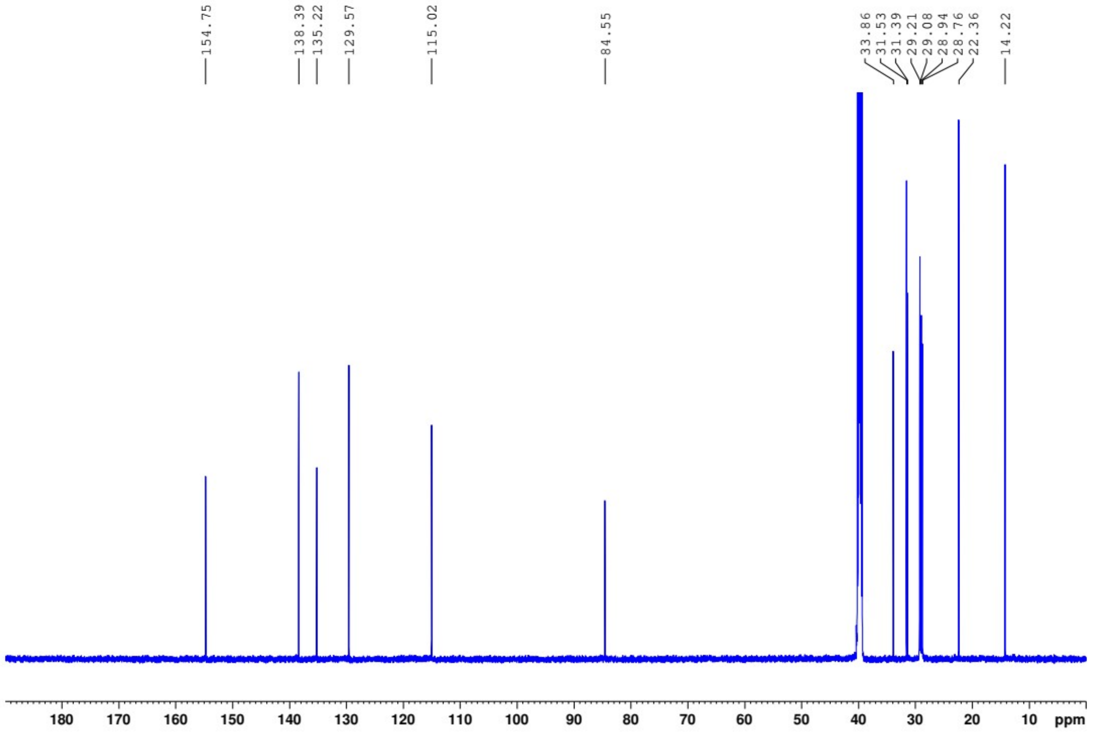

Figure S2: ^{13}C NMR spectrum of 2-iodo-4-nonylphenol in DMSO-*d6*

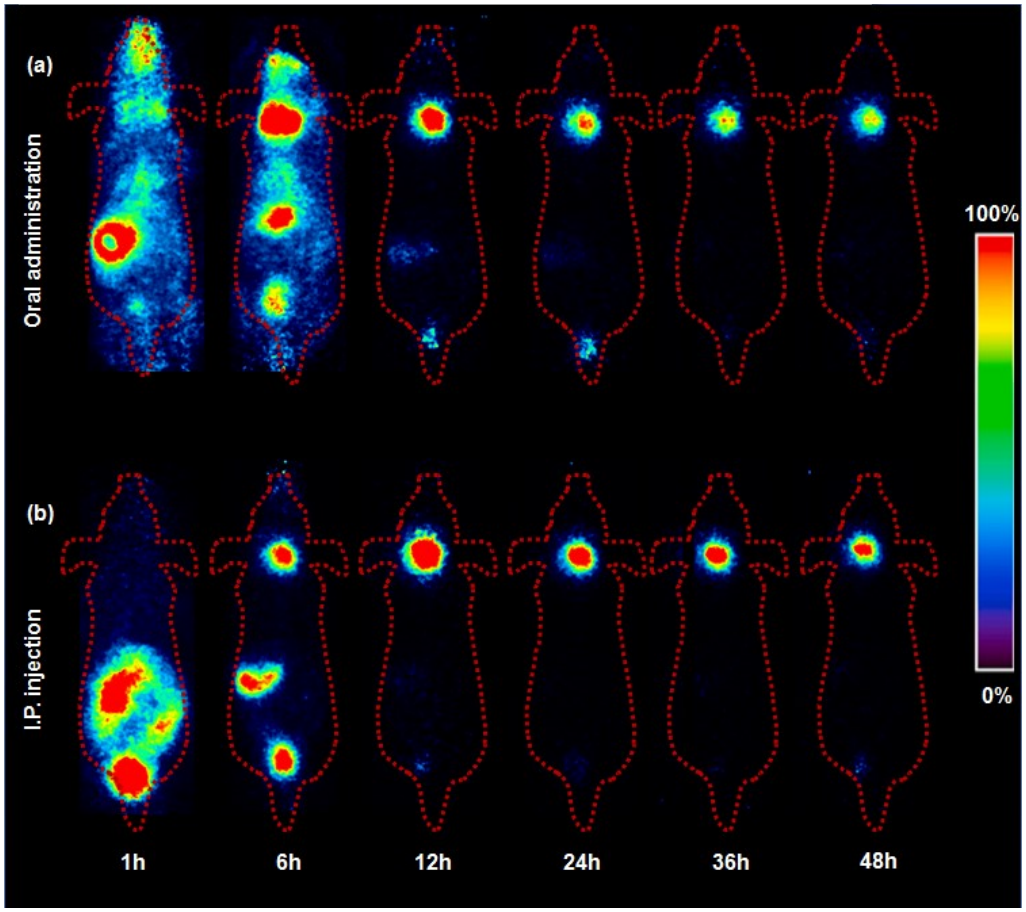

Figure S3. Representative PET images of free ^{124}I, acquired using 6-week-old ICR mice. (a) Oral and (b) intraperitoneal injection (n=3)

Table S1. The biodistribution data of ^{124}I-labeled 4NP after oral administration (n=4), data represented as %ID/g of organ or blood along with standard deviation values

	1hr	6hr	12hr	24hr	36hr	48hr
Blood	3.86 (0.34)	1.63(0.06)	0.09(0.03)	0.07(0.06)	0.06(0.08)	0.07(0.01)
Fat	0.59(0.89)	0.69(0.20)	0.70(0.01)	0.75(0.16)	0.68(0.04)	0.62(0.09)
Heart	2.06(0.10)	1.08(0.44)	0.04(0.01)	0.038(0.18)	0.05(0.03)	0.01(0.004)
Lung	3.43(0.47)	2.17(0.36)	0.09(0.04)	0.078(0.03)	0.08(0.06)	0.03(0.01)
Liver	3.59(0.24)	2.84(0.86)	0.14(0.01)	0.12(0.05)	0.12(0.01)	0.11(0.02)
Spleen	2.18(0.33)	1.25(0.48)	0.04(0.02)	0.045(0.01)	0.05(0.03)	0.03(0.01)
Stomach	55.86(2.97)	19.58(3.33)	1.54(0.12)	0.41(0.09)	0.04(0.006)	0.02(0.006)
L.Intestine	7.02(0.27)	15.86(2.19)	1.80(0.21)	1.38(0.13)	0.86(0.06)	0.33(0.05)
Kidney	4.31(0.48)	2.04(0.66)	0.08(0.02)	0.08(0.05)	0.16(0.04)	0.02(0.01)
Brain	0.19(0.02)	0.14(0.05)	0.01(0.001)	0.006(0.001)	0.007(0.004)	0.007(0.004)
S.Intestine	19.02(3.36)	6.62(1.65)	0.39(0.05)	0.15(0.04)	0.10(0.008)	0.01(0.003)
Thyroid	0.94(0.15)	0.78(0.43)	0.76(0.11)	0.22(0.31)	0.34(0.403)	0.41(0.15)

Table S2. The biodistribution data of ^{124}I-labeled 4NP after intraperitoneal injection (n=4), data represented as %ID/g of organ or blood along with standard deviation values

	1hr	6hr	12hr	24hr	36hr	48hr
Blood	4.57 (0.68)	1.73(0.32)	0.08(0.06)	0.07(0.08)	0.03(0.01)	0.02(0.01)
Fat	4.35(1.87)	4.62(2.75)	5.44(1.62)	7.42(1.16)	5.73(2.45)	4.42(1.38)
Heart	2.33(0.41)	0.79(0.07)	0.07(0.04)	0.07(0.06)	0.04(0.01)	0.03(0.02)
Lung	3.53(0.93)	1.53(0.14)	0.22(0.13)	0.23(0.15)	0.20(0.06)	0.20(0.39)
Liver	4.59(0.27)	1.84(0.62)	0.27(0.17)	0.26(0.21)	0.20(0.11)	0.11(0.01)
Spleen	3.79(1.60)	2.39(0.55)	0.30(0.24)	0.39(0.29)	0.43(0.28)	0.27(0.09)
Stomach	25.84(2.81)	19.77(2.46)	2.49(1.58)	1.23(1.16)	0.60(0.09)	0.32(0.06)
L.Intestine	5.72(1.58)	11.78(2.62)	1.53(0.84)	3.29(2.48)	0.65(0.08)	0.48(0.17)
Kidney	4.99(0.92)	3.04(1.67)	1.21(0.31)	0.61(0.62)	0.58(0.18)	0.59(0.41)
Brain	0.25(0.03)	0.14(0.02)	0.02(0.01)	0.02(0.01)	0.02(0.04)	0.01(0.005)
S.Intestine	9.87(2.52)	4.23(2.40)	2.07(0.51)	1.37(0.96)	0.70(0.39)	0.36(0.01)
Thyroid	1.57(0.36)	1.68(0.45)	2.32(0.16)	1.02(0.21)	0.92(0.35)	0.67(0.47)

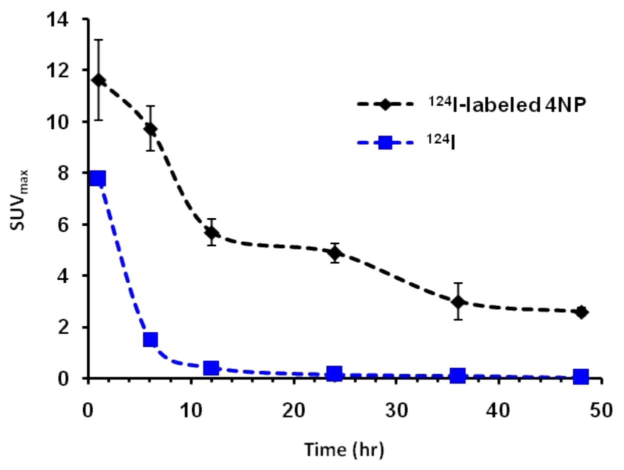

Figure S4. The maximum standard uptake values (SUV_{max}) of ^{124}I-labeled 4NP (black), and free radioiodine (^{124}I) (blue) in gastrointestinal tract after oral administration injection (n=3)

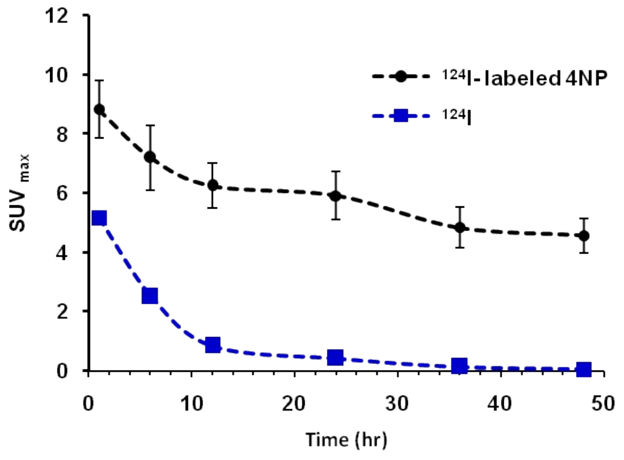

Figure S5. The maximum standard uptake values (SUV_{max}) of ^{124}I-labeled 4NP (black), and free radioiodine (^{124}I) (blue) in abdominal area after intraperitoneal injection (n=3)
